# Effects of Ultrasound-Guided Nerve Stimulation Targeting Peripheral Nerve Tissue on Pain and Function: A Scoping Review

**DOI:** 10.3390/jcm11133753

**Published:** 2022-06-28

**Authors:** Agustín García-Collado, Juan A. Valera-Calero, César Fernández-de-las-Peñas, José L. Arias-Buría

**Affiliations:** 1 Escuela Internacional de Doctorado, Universidad Rey Juan Carlos, 28922 Alcorcón, Spain; agustin.garciacollado@gmail.com; 2 Department of Physiotherapy, Faculty of Health, Universidad Camilo José Cela, 28692 Villanueva de la Cañada, Spain; 3 Department of Physical Therapy, Occupational Therapy, Rehabilitation and Physical Medicine, Universidad Rey Juan Carlos, 28922 Alcorcón, Spain; joseluis.arias@urjc.es

**Keywords:** percutaneous electrical nerve stimulation, nerve, pain, function, scoping review

## Abstract

This paper assesses the effects of percutaneous electrical nerve stimulation (PENS) on pain- and function-related outcomes by means of a scoping review of studies with single cases, case-series, quasi-experimental, and randomized or non-randomized trial designs. We consulted the PubMed, MEDLINE and EMBASE databases. Data were extracted by two reviewers. The methodological quality of studies was assessed using the Physiotherapy Evidence Database (PEDro) scale for experimental studies and the Joanna Briggs Institute (JBI) tool for case reports or cases series. Mapping of the results included: (1), description of included studies; (2), summary of results; and, (3), identification of gaps in the existing literature. Eighteen articles (five randomized controlled trials, one trial protocol, nine case series and three case reports) were included. The methodological quality of the papers was moderate to high. The conditions included in the studies were heterogeneous: chronic low back pain, lower limb pain after lumbar surgery, chronic post-amputation pain, rotator cuff repair, foot surgery, knee arthroplasty, knee pain, brachial plexus injury, elbow pain and ankle instability. In addition, one study included a healthy athletic population. Interventions were also highly heterogeneous in terms of sessions, electrical current parameters, or time of treatment. Most studies observed positive effects of PENS targeting nerve tissue against the control group; however, due to the heterogeneity in the populations, interventions, and follow-up periods, pooling analyses were not possible. Based on the available literature, PENS interventions targeting peripheral nerves might be considered as a potential therapeutic strategy for improving pain-related and functional outcomes. Nevertheless, further research considering important methodological quality issues (e.g., inclusion of control groups, larger sample sizes and comparatives between electric current parameters) are needed prior to recommending its use in clinical practice.

## 1. Introduction

Percutaneous electrical nerve stimulation (PENS) consists of the application of electric current through a solid filiform needle. The needle is inserted and ultrasound-guided until the tip of the needle is placed into musculoskeletal structures, but also nearby peripheral nerves to induce sensitive or motor stimulation with different therapeutic objectives [1]. This intervention is defined as a minimally invasive treatment and the US-guided use ensures patient safety with regard to avoiding adverse events derived from needling punctures of sensible tissues. Thus, it is a cost-effective intervention compared with pharmacological treatments or infiltrations [2]. The electrical current most commonly applied is biphasic, with different frequencies (ranging from 2–5 Hz or 80–100 Hz) and pulse widths (ranging from 250 to 500 ms), depending on the therapeutic objectives and effects desired [3,4].

It should be noted that there are several differences between PENS, neural PENS, TENS (transcutaneous electrical nerve stimulation) and electroacupuncture. For instance, TENS is characterized by the use of surface electrodes and no needles are used. However, its effectiveness has yet to be confirmed [5]. On the other hand, PENS targets peripheral nerves (neural PENS) or any other musculoskeletal structure to improve the patients’ symptomatology, while electroacupuncture is based on traditional Chinese medicine reasonings aiming specific points [4,5,6].

Although PENS was first described in 1952 [7], this therapeutic tool has been increasingly used for chronic pain management during the last 50 years [8,9], since this approach has been suggested to induce afferent input changes in the central nervous system (also known as the neuromodulation effect) [10,11,12,13,14,15,16,17]. In fact, previous studies have observed that PENS effectively reduced pain (either acute or chronic) [18,19,20] and also alleviate neuropathic pain conditions [16,21,22]. In addition, PENS has demonstrated other relevant applications, including the improvement of sports performance [17,23,24,25].

Scoping reviews are the most adequate method to examinate the current state of evidence regarding specific topics, to summarize the most relevant findings, to identify potential flaws providing novel guidelines for future research, to clarify concepts and to evaluate whether study designs are appropriate for future systematic reviews [26]. Therefore, it is a feasible alternative to other review designs (e.g., systematic reviews and meta-analyses) in those cases where reporting the meaningfulness or effectiveness of a therapeutic intervention is not possible [27]. Therefore, this scoping review aimed to map the existing literature regarding the effects of PENS targeting peripheral nerves on pain and function-related outcomes.

## 2. Methods

### 2.1. Study Design

This scoping review will provide the readers with a broad overview of the existent literature on PENS targeting peripheral nerves, where the heterogeneity of methods and populations could be comprised. As recommended by Arksey and O’Malley [28], we first identified the research question, identified relevant studies on this topic, selected the studies, charted the data and, finally, collated, summarized and reported the results extracted from the studies. We followed the guidelines reported on the Preferred Reporting Items for Systematic Reviews and Meta-Analyses Extension for Scoping Reviews (PRISMA-ScR) [29]. This scoping review was prospectively registered on 17 May 2022 in the Open Science Framework (registration DOI: 10.17605/OSF.IO/64RVM).

### 2.2. Identifying the Research Question

The research question aimed to analyze the potential clinical utility of PENS interventions aimed peripheral nerves for improving pain or functional outcomes. Therefore, the research question was: “Is Percutaneous Electrical Nerve Stimulation that targeting peripheral nerves an effective intervention for improving pain and related-functions?”

### 2.3. Identifying the Relevant Studies

A literature search was conducted on three databases as recommended by Dhammi and Haq [30] up to 31 May 2022 in the PubMed, MEDLINE and EMBASE databases. After a first scanning, we also revised those articles referenced in the identified papers. Since not all journals are indexed in those databases, we manually screened the articles published in specific key journals. The search was conducted by two members of the research group with the assistance of an experienced health science librarian. Articles were filtered to those published in the English or Spanish languages, conducted in humans and including single case studies, case-series, quasi-experimental, and randomized or non-randomized clinical trials.

The search strategy combined the following terms using Boolean operators follows for all databases as follows (Table 1).

### 2.4. Selecting the Studies

The PCC (Participants, Concept, Context) framework was followed to identify the main concepts:

Participants: Healthy participants or clinical populations with musculoskeletal pain.

Concept: Use of PENS targeting peripheral nerves.

Context: Evaluation of functional and pain-related changes after intervention

After a first screening, consisting of a first title and abstract reading, a full-text read of the remaining studies was conducted. In case of discrepancies between both reviewers, a third author would be asked to make a determination.

### 2.5. Charting the Data

Data extraction was conducted with a data charting form as recommended by Arkesy and O’Malley [28], providing a standardized summary of the results for each article included in the scoping review. All data were extracted by two authors including the authors’ information, year of publication, population, sample size, intervention details and pain or functional outcomes assessed [31]. Again, both authors had to achieve consensus on each item and in case of disagreement, a third author would provide a final decision.

### 2.6. Mapping the Data

After data extraction, we mapped the literature thematically, providing a description of the identified and included studies, a summary of the results and, finally, identifying gaps in the existing literature.

### 2.7. Methodological Quality Assessment

The methodological quality of all studies was assessed by both authors using the Physiotherapy Evidence Database (PEDro) scale [32] for experimental studies and the Joanna Briggs Institute (JBI) tool for case reports [33].

The PEDro scale is widely used to assess the methodological quality of experimental trials and includes 11 items. The first item, although is not included in the score, is related to external validity. The following 10 items are used to calculate the final score (ranging from 0 to 10 points), evaluating the random allocation, concealed allocation, similarity at baseline, subject blinding, therapist blinding, assessor blinding, lost follow-up, intention-to-treat analysis, between-group statistical comparison, and point/variability measures for at least one key outcome. Total scores between 0 and 3 are considered “poor”, 4 and 5 as “fair”, 6 and 8 are considered “good”, and 9 and 10 are “excellent” for this scale [32].

The methodological quality of case series and case reports was assessed using the JBI tool [33,34]. The critical appraisal of case reports assesses whether the studies describe the patient’s demographic characteristics, the patient’s history, the clinical condition of the patient, the diagnostic tests and results, the interventions, post-clinical conditions and adverse events, and if there is any key lesson learned from the exposed case, in an eight-item scale with Yes/No/Unclear possible answers for each item [33]. On the other hand, the JBI tool used for assessing the methodological quality of case series considers whether the studies described the inclusion criteria, if measurement tools were standard, valid and reliable, consecutive inclusion, completed inclusion of participants, reported the demographics and clinical information of participants, described the outcomes, presented the sites and clinics demographic information and statistical analyses were appropriate on a 10-point scale [34].

## 3. Results

### 3.1. Study Selection

**Figure 1:**
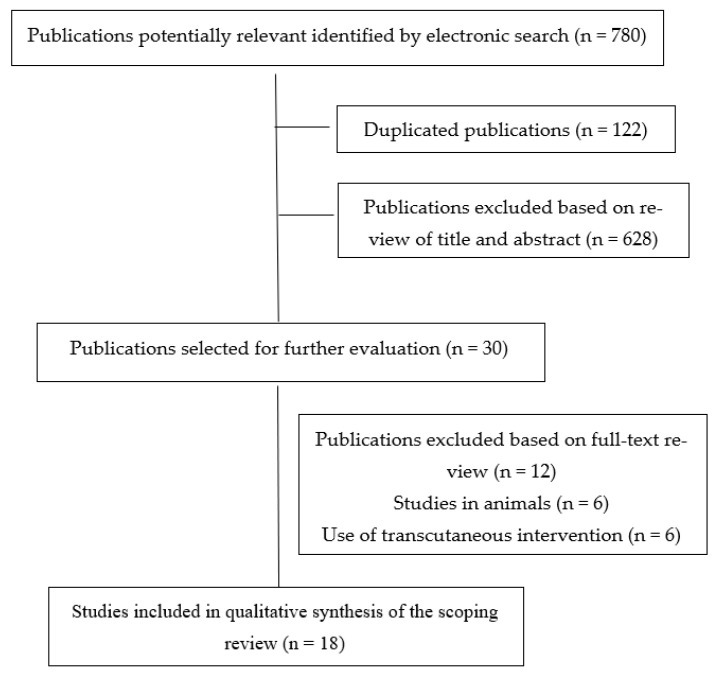
PRISMA Extension for Scoping Reviews (PRISMA-ScR) flow diagram.

Our electronic search resulted in 780 potential studies being included in this scoping review. After removing duplicates (n = 122) and those not meeting the first filter (n = 628), the full view text of 30 studies was conducted. After extensive reading, 12 studies were excluded. Therefore, a total of eighteen (n = 18) studies [10,11,12,35,36,37,38,39,40,41,42,43,44,45,46,47,48,49] were included in the literature data mapping (Figure 1).

### 3.2. Study Designs

All experimental studies were published in the last five years (from 2019). From six experimental studies, five were randomized controlled trials [11,36,37,47,49] and one was a trial protocol [12] reporting no results. Thus, nine studies were case series [10,35,39,41,42,43,45,46,48] and three articles described case reports [38,40,44].

### 3.3. Methodological Quality

The methodological quality assessment of the experimental studies is reported in Table 2. Scores ranged from 4 to 8 with a mean value of 6.5 ± 1.5 points. The most repeated flaw was the lack of intention to treat analysis [11,12,36,37,49]. On the other hand, all the studies considered a random allocation.

The methodological quality assessment of case reports is reported in Table 3. The only three case reports found during the review had a methodological quality score of 7 out of 8 points, this being the lack of adverse events description, which was the only flaw found in all of the studies [38,40,44,45].

Finally, the methodological quality assessment of case series is summarized in Table 4. All studies assessed the conditions with standard and reliable tools, used valid methods for identifying the condition, clearly reported the outcomes during the follow-up period and clearly reported the sites’ demographic information. The most constant flaw was the absence of consecutive inclusion of participants. In fact, only one study provided this information [43].

### 3.4. Summarizing Findings

The characteristics of the participants in the included studies are reported in Table 5. The total sample consisted of 257 patients (139 men, 128 women) with recruited samples ranging from single case reports [38,44] to clinical trials including 80 subjects [36].

Pain conditions were heterogeneous and included patients with chronic lower back pain [10,43,46], lower limb pain after lumbar surgery [38], chronic post-amputation pain [11], musculoskeletal impairments after unilateral rotator cuff repair [35], foot surgery [39] and knee arthroplasty [48], reduced hamstring flexibility [36], unilateral anterior knee pain [37,42], brachial plexus injury [40], lateral elbow pain [44] and ankle instability [45]. In addition, one study included a healthy athletic population [47].

Interventions were also heterogeneous in terms of sessions (Cohen et al. [10] programmed a 6-month intervention, 6 h per day while De-la-Cruz-Torres et al. [35] performed a single intervention of 1.5 min of duration), nerves targeted (medial branches of dorsal spinal ramus [10,43,46], femoral nerve [11,37,41,42,47,48], sciatic nerve [11,12,36,39,48], brachial plexus [12,35], peroneal nerve [38], radial nerve [40,44,49], tibial nerve [45]) and follow-up periods (ranging from post-intervention [42] to 2 years [44]). Regarding the electric parameters set among the studies, two therapeutic strategies could be differentiated. Half of the studies set approximately 100 Hz of frequency, 0.2 to 20 mA of amplitude and 15 to 200 μs pulse duration [10,11,35,39,41,48], while the other half set <10 Hz of frequency and 250 μs pulse duration [36,37,42,43,44,45,46,47,49].

Finally, the most assessed outcomes were pain intensity [10,11,12,35,37,38,39,40,41,43,44,46,48,49], range of movement [36,37,39,48], disability [43,44,46,49], medication intake [12,41,46], strength [42], stiffness [36], quality of life [40], body balance [45], morphological nerve changes [49] and sports performance [47]. Most studies observed positive effects of the intervention against the control group; however, due to the heterogeneity in the populations, interventions, and follow-up periods, pooling analyses were not possible.

## 4. Discussion

Although a previous meta-analysis analyzing the efficacy of PENS in pain-related outcomes has been published [5], this is the first scoping review focusing on pain and functional changes when PENS is specifically applied targeting the peripheral nerves.

### 4.1. Literature Mapping

Despite the short lifetime of this novel therapeutic approach, multiple pain conditions benefited from the use of PENS targeting peripheral nerve tissue. The most widely assessed conditions were chronic low back pain (three articles, all of them with a case series design [10,43,46]) and unilateral anterior knee pain (two articles, a randomized clinical trial [37] and a case series [42]). Even though one of the most important discussions is currently whether the frequency, duration, intensity and pulse width may induce different effects, none of the studies compared two different modalities of PENS in the same article.

Although previous studies have reported the mechanisms behind high-frequency and low-frequency currents (regarding the activation of endogenous opioid receptors) supporting the different peripheral antinociceptive responses depending on the stimulation received [5], three studies reported similar improvements in low back pain intensity and disability in the mid-term [43] and long-term [10,46]. However, it should be noted that previous studies analyzing the effects of electric current parameters on sensory variables were mostly focused on mechano-sensitivity indicators (e.g., pressure pain thresholds) instead of clinical self-reported pain intensity. While pain experience is highly subjective and a complex experience influenced by several factors [50], local responses dependent of electrical current parameters may produce changes in primary or secondary hyperalgesic areas [5].

Regarding the efficacy of PENS targeting peripheral nerves in populations with unilateral anterior knee pain, both studies used similar current parameters [37,42] and assessed pain-related (in the study conducted by García-Bermejo et al. [37]) and physical conditioning (in the study conducted by Álvarez-Prats et al. [42]) outcomes. In both cases, the studies showed significant changes compared with the baseline in terms of maximal isometric strength of the quadriceps, range of movement, pain intensity and disability in the short term.

Comparison between studies for the rest of the conditions including lower limb pain after lumbar surgery [38], chronic post-amputation pain [11], musculoskeletal impairments after unilateral rotator cuff repair [35], foot surgery [39] and knee arthroplasty [48], reduced hamstring flexibility [36], brachial plexus injury [40], lateral elbow pain [44] and ankle instability [45] was not possible as only one study was found for each condition. However, in general all studies reported good results in the short, middle and long term after PENS interventions targeting peripheral nerves.

Finally, the experimental studies including a comparative group were also heterogeneous. For instance, Garcia-Bermejo et al., [36] compared the effects of a single PENS intervention between clinical and healthy populations (with comparable effects between groups) while Gallego-Sendarrubias et al., [47] compared the inclusion of PENS as a complementary intervention to a training program in a sample of semiprofessional soccer players without symptoms. While the effects of PENS seem to be similar in both the clinical and healthy populations, the inclusion of this technique demonstrated additional improvements in the short-term regarding sports performance. However, in the mid-term these differences are not significant.

### 4.2. Strengths and Limitations of the Review

The results from this scoping review should be interpreted according to its potential strengths and limitations. Strengths of this scoping review include a comprehensive literature search, methodological rigor, data extraction, and the inclusion of studies (experimental studies, case reports and case series) of moderate to high methodological quality. However, some potential limitations are also present. First, despite the high number of studies initially identified, only a relatively small number (n = 17) were finally included in the review, since the remaining paper was a proposed protocol. The most important issue was the heterogeneity in the conditions, nerves targeted, outcomes and populations included. Second, most studies are from the same research teams. Third, most of the studies had a design with no control groups and limited samples, which limits the clinical application or the real effectiveness of this intervention. As has been previously stated, studies investigating the efficacy of PENS targeting different peripheral nerves, in different clinical conditions and assessing different outcomes (i.e., pain intensity, function, sports performance, muscle strength, muscle stiffness, range of movement or balance, for mentioning some), should be conducted to further elucidate whether this intervention could be recommended in specific conditions. In addition, further studies should consider the comparison between different current modalities in the short-, middle- and long-term in order to provide clinicians with guidelines based on adequate scientific evidence.

## 5. Conclusions

This scoping review analyzed the efficacy of PENS intervention targeting peripheral nerves on pain-related and functional outcomes in both clinical and asymptomatic populations. The results were highly heterogeneous in terms of conditions assessed, outcomes measured, follow-up periods, study designs, electric current parameters, samples, intervention programs, number of sessions and nerves targeted. Based on the available literature, PENS interventions targeting peripheral nerves might be considered as a potential therapeutic strategy for improving pain-related and functional outcomes. Nevertheless, further research considering important methodological quality issues (e.g., inclusion of control groups, larger sample sizes and comparatives between electric current parameters) are needed prior to recommending its use in clinical practice.

## Figures and Tables

**Table 1:** Database formulas during literature search.

**PubMed Search Formula** #1 “Ultrasound guided percutaneous neuromodulation” [Title/Abstract] OR “Percutaneous Electrical Nerve Stimulation” [Title/Abstract] OR “Nerve Tissue Stimulation” [Title/Abstract] #2 “Pain” [Mesh] OR “Related-disability” [Title/Abstract] OR “Function” [Title/Abstract] OR “Mobility” [Title/Abstract] #3 #1 AND #2
**Medline (via EBSCO) Search Formula** #1 “Ultrasound guided percutaneous neuromodulation” OR “Percutaneous Electrical Nerve Stimulation” OR “Nerve Tissue Stimulation” #2 “Pain” OR “Related-disability” OR “Function” OR “Mobility” #3 #1 AND #2
**WOS (EMBASE, AMED) Search Formula** (“Ultrasound guided percutaneous neuromodulation” OR “Percutaneous Electrical Nerve Stimulation” OR “Nerve Tissue Stimulation”) AND (“Pain” OR “Related-disability” OR “Function” OR “Mobility”)

**Table 2:** Physiotherapy Evidence Database (PEDro) scale for assessing the methodological quality of the studies included.

Reference	Study Type	PEDro Scale Items											Score
		1	2	3	4	5	6	7	8	9	10	11	
Ilfeld et al., 2019 [11]	RCT	+	+	+	+	+	-	+	+	-	+	+	8
Ilfeld et al., 2020 [12]	RCT-P	+	+	−	−	+	+	+	-	-	-	-	4
De-la-Cruz-Torres et al., 2021 [36]	RCT	+	+	+	+	-	-	+	+	-	+	+	7
García-Bermejo et al., 2020 [37]	RCT	+	+	-	+	-	-	+	+	-	+	+	6
Gallego-Sendarrubias et al., 2021 [47]	RCT	+	+	+	+	-	-	+	+	+	+	+	8
De-la-Cruz-Torres et al., 2021 [49]	RCT	+	+	-	+	-	-	+	+	-	+	+	6

RCT: Randomized Clinical Trial; RCT-P: Randomized Clinical Trial Protocol. 1: selection criteria; 2: random allocation; 3: concealed allocation; 4: similarity at baseline; 5: subject blinding; 6: therapist blinding; 7: assessor blinding; 8: >85% measures for initial participants; 9: intention to treat; 10: between-group statistical comparisons; 11: point and variability measures. None of the selected articles had a conflict of interest; −: No; +: Yes.

**Table 3:** JIB tool for assessing the methodological quality of case reports.

Reference	JBI’s Tool for Assessing Case Series							
	1	2	3	4	5	6	7	8
Ferreira-Dos-Santos et al., 2019 [38]	Y	Y	Y	Y	Y	Y	N	Y
Kim et al., 2017 [40]	Y	Y	Y	Y	Y	Y	N	Y
Arias-Buría et al., 2019 [44]	Y	Y	Y	Y	Y	Y	N	Y

(1) Were patient’s demographic characteristics clearly described ?; (2) Was the patient’s history clearly described and presented as a timeline?; (3) Was the current clinical condition of the patient on presentation clearly described?; (4) Were diagnostic tests or assessment methods and the results clearly described ?; (5) Was the intervention(s) or treatment procedure(s) clearly described?; (6) Was the post-intervention clinical condition clearly described?; (7) Were adverse events (harms) or unanticipated events identified and described?; 8: Does the case report provide takeaway lessons? N: No; Y: Yes.

**Table 4:** JIB tool for assessing the methodological quality of case series.

Reference	JBI’s Tool for Assessing Case Series									
	1	2	3	4	5	6	7	8	9	10
Cohen et al., 2019 [10]	Y	Y	Y	N	Y	Y	Y	Y	Y	Y
Ilfeld et al., 2019 [35]	Y	Y	Y	N	Y	Y	Y	Y	Y	Y
Ilfeld et al., 2018 [39]	Y	Y	Y	N	Y	Y	Y	Y	Y	Y
Ilfeld et al., 2019 [41]	Y	Y	Y	N	Y	Y	Y	Y	Y	Y
Álvarez-Prats et al., 2019 [42]	Y	Y	Y	N	Y	Y	Y	Y	Y	Y
Sanmartin-Enriquez et al., 2019 [43]	N	Y	Y	Y	Y	N	N	Y	Y	N
Rodríguez-Rosal et al., 2019 [45]	N	Y	Y	N	Y	Y	Y	Y	Y	Y
Gilmore et al., 2019 [46]	Y	Y	Y	N	Y	Y	Y	Y	Y	Y
Ilfeld et al., 2017 [48]	Y	Y	Y	N	Y	Y	Y	Y	Y	Y

(1) Were there clear criteria for inclusion in the case series?; (2) Was the condition measured in a standard, reliable way for all participants included in the case series?; (3) Were valid methods used for identification of the condition for all participants included in the case series?; (4) Did the case series have consecutive inclusion of participants?; (5) Did the case series have complete inclusion of participants?; (6) Was there clear reporting of the demographics of the participants in the study?; (7) Was there clear reporting of clinical information of the participants?; (8) Were the outcomes or follow-up results of cases clearly reported?; (9) Was there clear reporting of the presenting sites’/clinics’ demographic information?; (10) Was statistical analysis appropriate? N: No; Y: Yes; U: Unclear.

**Table 5:** Data extraction of the studies included in the scoping review.

Reference	Population	Intervention	Outcomes	Results Summary
Álvarez-Prats et al., 2019 [42]	13 Subjects with history of unilateral knee pathology and were in the stage of recovery of quadriceps strength. 11 males 2 females	Target: Femoral nerve Description: A single intervention consisting of 10 stimulations with a duration of 10 s, with a 10-s rest period between each stimulation	Pre- and post-intervention Quadriceps dynamometry	Significant changes were obtained in the maximal isometric strength of the quadriceps of the affected knee
Arias-Buría et al., 2019 [44]	1 Male with lateral elbow pain	Target: Radial nerve Description: 2 sessions of ultrasound-guided PENS and 4-weeks of a low-load concentric-eccentric exercise program of the wrist extensors	2 years follow-up Pain intensity (Numeric Pain Rate Scale), function (Patient-Rated Tennis Elbow 45 Evaluation), and related disability (Disabilities of the Arm, Shoulder and Hand Outcome Measure)	The patient progressively exhibited complete resolution of pain and function, which was maintained at 2 years
Cohen et al., 2019 [10]	9 Subjects with chronic low back pain 1 male 8 females	Target: Medial branches of the dorsal primary ramus Description: 1 month of duration, 6 h per day Single group	7 months follow-up Disability (Oswestry Disability Index), Pain Interference (BPI-9) Patient Global Impression of Change, Pain Intensity (BPI-5), Analgesics intake	The intervention induced significant reductions in pain intensity, disability, pain interference and medication intake from the first month to the seventh month compared with baseline
De-la-Cruz-Torres et al., 2021 [36]	80 participants with reduced hamstring flexibility 40 males 40 females	Target: Sciatic nerve Description: A single intervention of 1.5 min PENS single intervention versus stretching, neurodynamic and dry needling	Pre- and post-intervention assessment Bilateral straight leg raise test Tensiomyography	The PENS and needle groups obtained higher values for the SLR test in the non-intervention limb compared with the other groups. There were statistically significant differences for mean SLR measures between limbs pre- and post-intervention for all groups except the PENS group, suggesting crossover effects for PENS but not the other techniques studied. No differences in tensiomyographic assessments between groups or between sides were seen.
De-la-Cruz-Torres et al., 2021 [49]	24 Subjects with chronic lateral Epicondylalgia 12 males 12 females	Target: Radial nerve Description: A single intervention of PENS vs. no intervention	1 month follow-up Pain intensity, functionality, electrophysiologic excitability, and nerve morphology	After 1 month, PENS group improved their values compared to their baseline values (pain intensity and nerve cross-sectional area values showed a significant decrease while the patient-rated tennis elbow evaluation scores showed a significant improvement. Thus, the PENS group showed significant improvement for the electrophysiologic nerve excitability pattern, reporting normal function in all radial nerves after treatment. However, chronaxie values always reported similar values with no differences between groups
Ferreira-Dos-Santos et al., 2019 [38]	A single male case with a medical history significant for multiple lumbar surgeries with multiple complications	Target: Superficial peroneal nerve Description: The treatment duration was 3 months	3 months follow-up Pain intensity	Two weeks after implantation of the percutaneous PENS, the patient reported he was walking 5 times farther than his typical morning walk and experienced a reduction of pain from 8 to 1 in the numeric rating scale. After 3 months, the patient reported maintenance of improvements.
Gallego-Sendarrubias et al., 2021 [47]	23 Semiprofessional male soccer players	Target: Femoral nerve Description: One group received a training program while the other group received two PENS interventions.	1-month follow-up Countermovement jump and squat performance speed	Male soccer players receiving PENS intervention before the training session experienced greater increases in flight time, and in vertical jump height, after both sessions, but not one month after than those who did not receive PENS intervention. Similarly, soccer players receiving the PENS intervention experienced a greater increase in the squat performance speed after the second session, but not after the first session or one month after the intervention
García-Bermejo et al., 2020 [37]	28 Subjects with Unilateral Anterior Knee Pain 14 males 16 females (It should be noted that 2 participants withdrawn the study and the authors did not report the gender)	Target: Femoral nerve Description: A single intervention of 1.5 min Asymptomatic and patients with pain received the same PENS intervention	1-week follow-up Numeric rating score, range of motion, Kujala and Victorian Institute of Sport Assessment-Patella	Both groups showed an increase immediately at 24 h, and at 1 week for the knee flexion ROM variable. The symptomatic knee group showed an increase for the Kujala score and a decrease for the numeric rating scale (NRS) variable from baseline to 1 week. VISA-P score did not show significant differences. After the intervention, there were no differences between the groups in any measured time
Gilmore et al., 2019 [46]	9 Subjects with chronic low back pain 1 male 8 females	Target: Medial branch of the dorsal ramus Description: All subjects received the same PENS intervention. Percutaneous fine-wire leads remained in place for the duration of the 30-day therapy	4 months follow-up Medication intake, disability (Oswestry Disability Index), pain interference (BPI-9), patient global impression of change	Most subjects reported significant reductions in pain intensity with PENS treatment, which continued four-months after. Subject also reported concomitant reductions in analgesic medication usage and significant reductions in patient-centric outcomes of disability, pain interference, and patient global impression of change.
Ilfeld et al., 2017 [48]	5 Subjects with history of total knee arthroplasty 2 males 3 females	Target: Femoral and sciatic nerves Description: All subjects received the same PENS intervention	ON-OFF comparison Pain intensity at rest and passive and active knee motion and range of movement	Percutaneous peripheral nerve stimulation decreased pain an average of 93% at rest, with 4 of 5 subjects experiencing complete resolution of pain. During passive and active knee motion pain decreased an average of 27 and 30%, respectively. Neither maximum passive nor active knee range-of-motion was consistently affected.
Ilfeld et al., 2018 [39]	7 Subjects undergoing ambulatory foot surgery 1 male 6 females	Target: Sciatic nerve Description: Subjects received 5 min of either stimulation or sham in a randomized, double-masked fashion followed by a 5-min crossover period and then continuous stimulation until lead removal on postoperative days 14 to 28	28 days follow-up Pain intensity, sensory deficits and the ability to move the ipsilateral great toe	During the initial 5-min treatment period, the stimulation induced a downward trajectory in their pain over the 5 min of treatment, whereas sham intervention resulted in no such change until their subsequent 5-min stimulation cross- over. During the subsequent 30 min of stimulation, pain scores decreased to 52% of baseline.
Ilfeld et al., 2019 [11]	26 Subjects with chronic postamputation pain 3 males 23 females	Target: Femoral and sciatic nerves Description: 2 months Placebo group and Intervention group crossed after 4 weeks	12 months follow-up Average residual limb pain Phantom limb pain	A significantly greater proportion of subjects receiving PNS (58%) demonstrated ≥50% reductions in average postamputation pain during weeks 1–4 compared with subjects receiving placebo (14%). Significantly greater proportions of PENS subjects also reported ≥50% reductions in pain (67%) and pain interference (80%) after 8 weeks of therapy compared with subjects receiving placebo (pain: 14%; pain interference: 15%).; Four of five PNS subjects who have completed 12-month follow-up to date reported ≥50% pain relief.
Ilfeld et al., 2019 [35]	14 subjects following unilateral rotator cuff repair 12 males 2 females	Target: Brachial plexus (5 posterior to the superior brachial plexus trunk, 6 adjacent to the C5 nerve root, and 3 posterior to the distal middle trunk) Description: Subjects received 5 min of either stimulation or sham in a randomized, double-masked fashion followed by a 5 min crossover period, and then continuous stimulation until lead removal postoperative days 14–28 PENS intervention versus placebo	90 days follow-up Average and maximum pain at rest Average and maximum pain with movement Opioids consumption	Stimulation did not decrease pain scores during the first 40 min of the subjects with brachial plexus leads, regardless of which treatment subjects were randomized to initially. Seven subjects required a single-injection interscalene nerve block for rescue analgesia prior to discharge. However, subsequent average resting and dynamic pain scores postoperative days 1–14 had a median of 1 or less on the Numeric Rating Scale, and opioid requirements averaged less than 1 tablet daily with active stimulation.
Ilfeld et al., 2019 [41]	10 Subjects with Ambulatory Anterior Cruciate Ligament Reconstruction 5 males 5 females	Target: Femoral nerve Description: Subjects received 5 min of either stimulation or sham followed by a 5-min crossover period, and then continuous active stimulation until lead removal postoperative Day 14–28.	3 months follow up Medication intake and pain intensity (at rest and with movement, average and maximum intensity)	During the initial 5-min treatment period, subjects randomized to stimulation experienced a decrease of 7% in their pain over the 5 min of treatment, while those receiving sham reported a slight increase of 4% until their subsequent 5-min stimulation crossover, during which time they also experienced a decrease of 11% from baseline. The median daily opioid consumption was less than 1 tablet.
Ilfeld et al., 2020 [12]	Subjects with postoperative pain after rotator cuff repair, hallux valgus correction, and ankle arthrodesis or arthroplasty	Target: Brachial plexus (shoulder) Sub-gluteal sciatic nerve (foot and ankle) Description: 14 days PENS intervention versus interscalene (shoulder) or a popliteal sciatic (foot and ankle) nerve block with ropivacaine 0.5% and epinephrine	12 months follow-up Opioid consumption Surgical pain Physical and emotional functioning	This is a protocol and therefore no results are currently reported
Kim et al., 2017 [40]	2 Males with severe neuropathic pain after incomplete brachial plexus injury	Target: Radial nerve	1 year follow-up Pain intensity and sleep and life quality	Their pain was relieved by more than 50% over the course of 1 year. Both patients were satisfied with their improved sleep and quality of life
Rodríguez-Rosal et al., 2019 [45]	5 Males with chronic ankle instability	Target: Tibial nerve Description: The process was performed on three occasions during 30 s, with an intensity that was acknowledged by the patient but which did not go beyond a score of 3 in the visual analog scale	The duration was not reported Body balance (The displacement of the center of pressure was determined based on the distances of its antero-posterior axes and medio-lateral. The amplitudes of anteroposterior and medio- lateral displacement were also evaluated)	A decrease was found in the antero-posterior amplitude
Sanmartin-Enriquez et al., 2019 [43]	10 Subjects with non- radiating low back pain 5 males 5 females	Target: Medial branch of a L2 posterior ramus and the iliohypogastric and ilioinguinal nerves Description: All subjects received 3 sessions, once a week	3 weeks follow-up Lumbar disability and pain intensity	80% of patients improved after the treatment protocol. A decrease in activity limitations was observed, from 14 to 4.35/1000 points on the Oswestry questionnaire, and a decrease of 6.8 to 2.15/10 points was observed on the Visual Analogue Scale.

PENS: Percutaneous Electrical Nerve Stimulation.

## Data Availability

All data derived from this study is reported in this article.
